# Association of tumor deposits with tumor-infiltrating lymphocytes and prognosis in gastric cancer

**DOI:** 10.1186/s12957-022-02507-3

**Published:** 2022-02-27

**Authors:** Xinyue Li, Jing Yang

**Affiliations:** 1 Pathology Department of the First Affiliated Hospital of Jinzhou Medical University, Jinzhou, Liaoning China; 2 Pathology Department of Jinzhou Medical University, Jinzhou, Liaoning China

**Keywords:** Tumor deposits, Tumor-infiltrating lymphocytes, Prognosis

## Abstract

**Background:**

To investigate the relationship between tumor deposits (TDs) with the clinicopathological characteristics tumor-infiltrating lymphocytes (TILs) and prognosis of gastric cancer. Further analysis was done on the relationship between the number and maximum diameter of TDs with the clinicopathological characteristics and prognosis of gastric cancer.

**Methods:**

The pathological findings of 369 patients with gastric cancer were retrospectively analyzed to observe the expression of TDs and the levels of stromal TILs. The relationship between TDs, clinicopathological characteristics, and levels of stromal TILs was compared using the chi-square test. Kaplan-Meier was used for survival analysis, and the log-rank test was used to determine the relationship between TDs and disease-free survival, cancer-specific survival, and overall survival. The prognostic value of TDs was assessed using multivariate Cox proportional hazards regression analysis. For further analysis, the optimal cutoff values for the number and maximum diameter of TDs were selected based on the receiver operating characteristic (ROC) curve.

**Results:**

TDs were significantly associated with sex, lymphovascular invasion, perineural invasion, pathological T,N stage, and clinical stage (all *P* < 0.05). TILs levels are lower in TDs(+) group and higher in TDs(−) group. Compared with TDs(−) groups, TDs(+) group had poor disease-free survival, cancer-specific survival, and overall survival. TDs are negatively correlated with TILs, and TILs levels are lower in TDs(+) group and higher in TDs(−) group (*P* < 0.05). The samples are divided into the number of TDs (< 4 and ≥ 4) and the maximum diameter of TDs (< 7 mm and ≥ 7 mm). The number of TDs was significantly associated with pathological N stage (*P* < 0.05). The maximum diameter of TDs was significantly correlated with Lauren classification (*P* < 0.05) .TDs ≥ 4 had lower DFS, CSS, and OS (*P* < 0.05). The maximum diameter of TDs was not statistically significant with prognosis (*P* > 0.05).

**Conclusion:**

TDs are independent prognosis predictors of gastric cancer. In the tumor microenvironment, TDs and TILs interact with each other to regulate the development of gastric cancer, thus affecting gastric cancer prognosis of patients. The number of TDs ≥ 4 has a worse prognosis compared to the number of TDs < 4.

## Introduction

Gastric cancer is an important cancer worldwide, with the fourth highest mortality rate [1]. At present, the prognosis of gastric cancer patients and the formulation of treatment plans mainly rely on clinicopathological parameters and molecular indicators [2, 3], but due to the heterogeneity of gastric cancer, none of these indicators can fully accurately reflect the prognosis of gastric cancer patients. Thus, it is especially important to study and enrich new prognostic indicators. TDs were firstly described as colorectal cancer mesenteric satellites in 1935 [4], and according to the American Joint Cancer Committee (AJCC) 8th TNM staging system for colorectal cancer, TDs were clearly defined as discrete tumor nodules within the lymphatic drainage area of primary cancer and without identifiable lymph node tissue or identifiable vascular or neural structures [5]. In colorectal cancer, TDs were included in staging treatment because they have been shown to be an independent prognostic factor [6, 7]. However, TDs have not been included in the pathological staging of gastric cancer due to limited research evidence. Only a few studies in the literature have found TDs to be strongly associated with a poor prognosis in gastric cancer [2, 8, 9]. In addition, tumor immune response has gradually become a hot issue in recent years as the study of tumor microenvironment has been intensified. However, the relationship between tumor deposition and tumor microenvironment has been rarely reported; tumor-infiltrating lymphocytes, as an important component of tumor microenvironment, were an important mechanism for the body to cope with tumor cells and induce tumor immune response [10, 11]. Therefore, this study aimed to investigate the relationship between TDs, TILs, and prognosis, to provide new ideas for the diagnosis and treatment of gastric cancer.

## Materials and methods

### Clinical information

Clinicopathological data were collected from January 2016 to December 2019 from patients who underwent surgical resection of gastric cancer at The First Affiliated Hospital of Jinzhou Medical University. The inclusion criteria are as follows: (1) patients with pathologically confirmed gastric cancer, (2) patients who had not received preoperative neoadjuvant radiotherapy or other adjuvant treatment, and (3) patients with complete clinical information and follow-up information. The exclusion criteria are as follows: (1) patients lost follow-up, (2) those with other malignancies, and (3) those with a preoperative co-infection or autoimmune disease. This study was approved by the Ethics Committee of The First Affiliated Hospital of Jinzhou Medical University (ethics number: KYLL 202029).

### Interpretation of TDs

Two independent pathologists separately reviewed pathological sections of gastric cancer using a double-blind method, and disagreements were confirmed by a third expert. TDs were defined as discrete tumor nodules within the lymph drainage area of the primary carcinoma without identifiable lymph node tissue or identifiable vascular or neural structure [5].

### Interpretation of TILs

The two independent pathologists assessed the percentage of stromal TILs in the central tumor and invasive margin of gastric cancer foci using a double-blind method and the assessment methods recommended by the 2014 International Working Group on tumor-infiltrating lymphocytes [12]. The methods for interpretation were as follows: (1) determine the extent of TILs assessment (TILs within the tumor border, including border locations, were assessed, and extratumoral and intraepithelial TILs, peri-tumoral tertiary lymphatic structures, and extensive necrosis or fibrosis were not assessed); (2) target the area of stromal TILs in the tumor (intraepithelial TILs were not assessed); (3) scan the entire field of view at low magnification; (4) determine the type of infiltrating cells, and count only single nucleated cells (lymphocytes and plasma cells); and (5) derive a percentage based on the ratio of the area occupied by stromal TILs to the total area of the interstitium, from which two groups were classified: a group with low-to-medium TILs, with a percentage of < 40%, and a group with high TILs, with a percentage of 40–90%. Averages were taken after the evaluation of multiview observations and were not focused on the hotspot view with the most infiltration.

### Follow-up information

Follow-up of the entire population was measured from the date of surgery to the time of last follow-up (September 2020) or date of all-cause death, and the median follow-up time was 27 (1–54) months, with reviews per month for 3 years post-surgery, every 6 months for 3–5 years post-surgery. Follow-up information was obtained by telephonic follow-up. Disease-free survival (DFS) is defined as the time from the start of follow-up until disease recurrence, metastasis, or progression; cancer-specific survival (CSS) is defined as the time from the start of follow-up to death due to gastric cancer; and overall survival (OS) is defined as the time from the start of follow-up to the patient’s death due to any other cause.

### Statistical processing

All data were statistically analyzed using the SPSS 21.0 software (IBM Corporation, Armonk, NY, USA). The relationships between TDs status, clinicopathological characteristics, and TILs infiltration level were compared using the chi-square test, and rank data were tested using the rank-sum test. Kaplan-Meier was used for survival analysis, and the log-rank test was used to determine the differences in survival curves between groups. The prognostic value of TDs was assessed using multivariate Cox proportional hazards regression analysis. *P* < 0.05 indicated a statistically significant difference.

## Results

### Relationships between tumor deposits and the clinicopathological characteristics

**Fig. 1:**
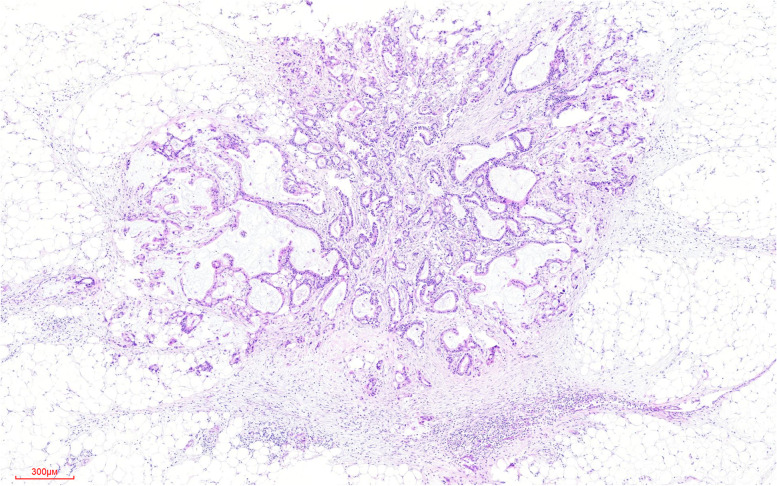
An example of a tumor deposit (TD) of gastric cancer to show the pathological features. Tumor deposits (TDs) were clearly defined as discrete tumor nodules within the lymphatic drainage area of primary cancer and without identifiable lymph node tissue or identifiable vascular or neural structures

Of the 369 gastric cancer cases, 81 had TDs+ (22.0%) (Fig. 1). TDs were significantly associated with sex, lymphovascular invasion (LVI), perineural invasion (PNI), pathological T,N stage, and clinical stage (all *P* < 0.05), whereas there was no statistical difference between age, histologic grade, Lauren classification, and mismatch repair gene (MMR) (all *P* > 0.05) (Table 1). For further analysis, the optimal cutoff values for the number and maximum diameter of TDs were selected based on the receiver operating characteristic (ROC) curve, and the samples were divided into the number of TDs (< 4 and ≥ 4) and the maximum diameter of TDs (< 7 mm and ≥ 7 mm). The number of TDs was significantly associated with pathological N stage (*P* < 0.05). The maximum diameter of TDs was significantly correlated with Lauren classification (*P* < 0.05).

**Table 1:** Association between tumor deposits clinicopathologic characteristics of gastric cancer.

	TDs+	TDs−	*P*
Sex			
Male	65	196	0.033
Female	16	92	
Age, years			
≤ 60	27	98	0.907
> 60	54	190	
Histologic grade			
Undifferentiated	69	224	0.145
Differentiated	12	64	
Lauren classification			
Diffuse type	41	152	0.241
Mixed type	27	72	
Intestines type	13	64	
LVI			
Yes	77	171	0.000
No	4	117	
PNI			
Yes	71	152	0.000
No	10	136	
MMR			
pMMR	76	253	0.126
dMMR	5	35	
pT stage			
T1	0	60	0.000
T2	2	46	
T3	14	80	
T4	65	102	
pN stage			
N0	2	102	0.000
N1	4	64	
N2	14	51	
N3	61	71	
pM stage			
M0	74	288	0.000
M1	7	0	
Clinical stage			
I	0	73	0.000
II	6	88	
III	68	127	
IV	7	0	

*LVI* lymphovascular invasion, *PNI* perineural invasion, *MMR* mismatch repair gene, *pMMR* proficient mismatch repair, *dMMR* deficient mismatch repair, *TDs* tumor deposits

### Relationships between tumor deposits and the prognosis of gastric cancer

**Fig. 2:**
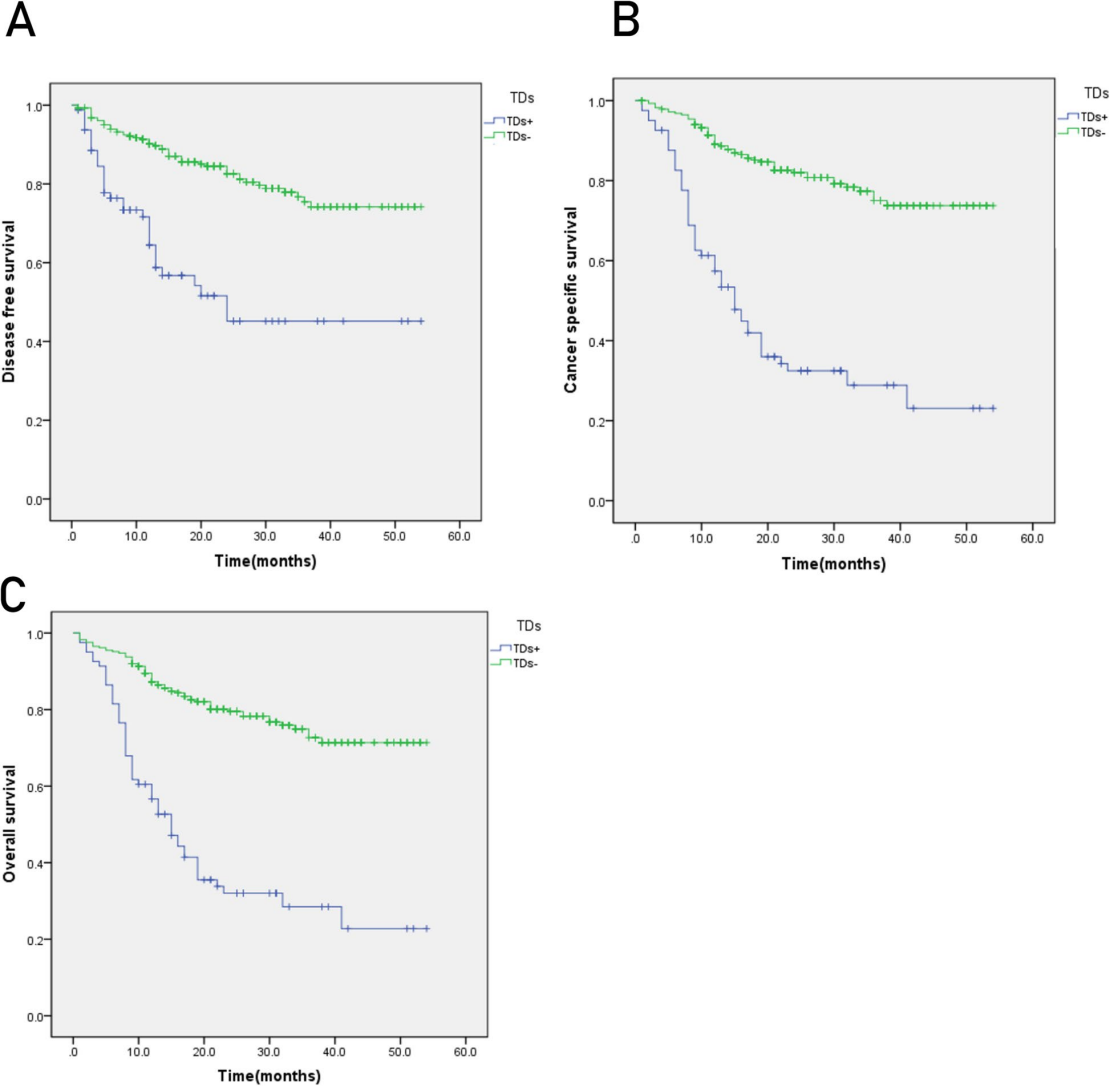
Kaplan-Meier curves for tumor deposits. **A** Disease-free survival (DFS). **B** Cancer-specific survival (CSS). **C** Overall survival (OS). *P* < 0.05

**Fig. 3:**
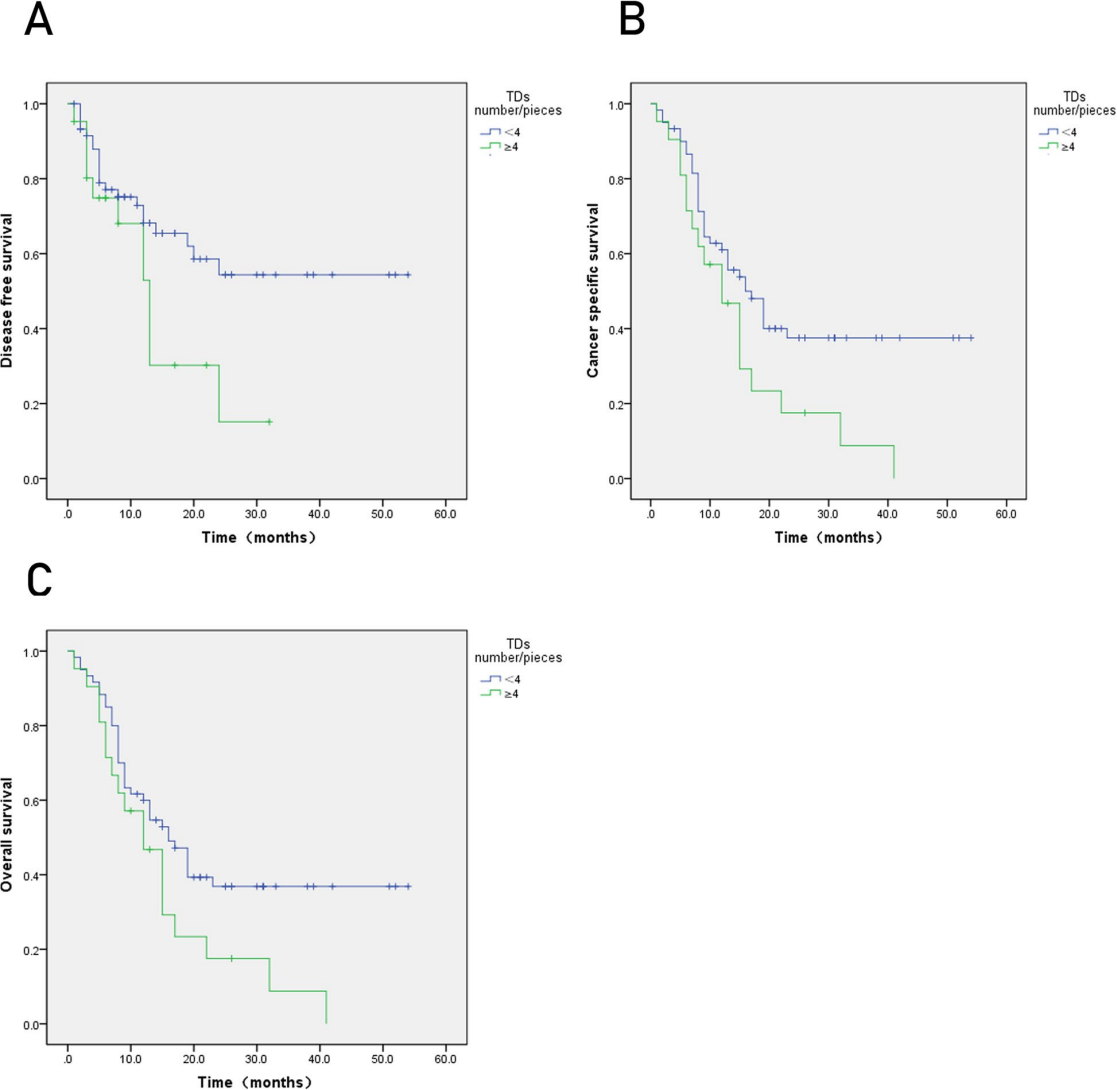
Kaplan-Meier curves for number of tumor deposits. **A** Disease-free survival (DFS). **B** Cancer-specific survival (CSS). **C** Overall survival (OS). *P* < 0.05

**Fig. 4:**
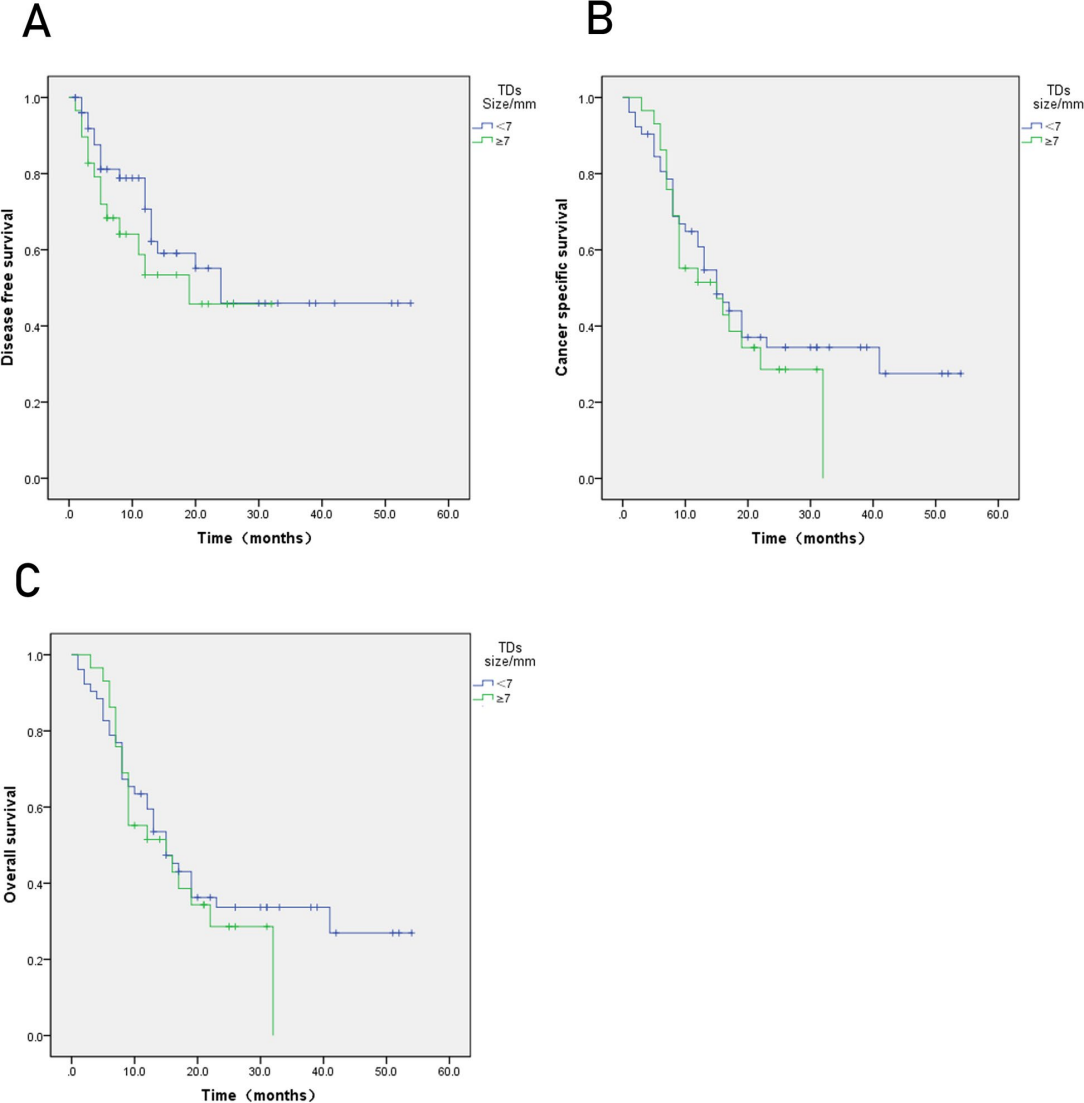
Kaplan-Meier curves for size of tumor deposit. **A** Disease-free survival (DFS). **B** Cancer-specific survival (CSS). **C** Overall survival (OS). *P* > 0.05

The TDs+ group had lower DFS, CSS, and OS compared to the TDs(−) group (Fig. 2), and TDs were an independent prognostic factor for DFS, CSS, and OS (Table 2). TDs ≥ 4 had lower DFS, CSS, and OS (*P* < 0.05) (Fig. 3). The maximum diameter of TDs was not statistically significant with prognosis (*P* > 0.05) (Fig. 4).

**Table 2:** Cox multivariate regression analysis of disease-free survival, cancer-specific survival, and overall survival correlation of tumor deposits

	DFS		CSS		OS	
	HR (95% CI)	*P*-value	HR (95% CI)	*P*-value	HR (95% CI)	*P*-value
TDs	0.571 (0.353–0.923)	0.022	0.464 (0.302–0.713)	0.000	0.538 (0.356–0.814)	0.003
Age, years	–	–	–	–	0.638 (1.089–2.462)	0.018
Histologic grade	–	–	1.040 (0.444–2.433)	0.929	1.198 (0.540–2.659)	0.657
Lauren classification	–	–		0.610		0.446
Lauren (1)			0.668 (0.268–1.663)	0.385	0.579 (0.248–	0.207
Lauren (2)			0.863 (0.311–2.399)	0.778	1.354) 0.688 (0.264–1.792)	0.444
LVI	0.549 (0.207–1.454)	0.228	0.960 (0.392–2.348)	0.929	0.940 (0.404–2.184)	0.885
PNI	0.511 (0.259–1.009)	0.053	0.599 (0.324–1.105)	0.101	0.685 (0.387–1.214)	0.195
pT stage		0.353		0.019		0.009
T(1)	0.703 (0.119–4.158)	0.698	0.290 (0.021–3.940)	0.352	1.023 (0.162–6.454)	0.981
T(2)	0.641 (0.091–4.519)	0.655	2.000 (0.134–29.774)	0.615	2.879 (0.312–26.583)	0.351
T(3)	1.070 (0.144–7.925)	0.947	4.142 (0.269–63.863)	0.309	6.655 (0.700–63.284)	0.099
pN stage		0.590		0.126		0.078
N(1)	0.921 (0.274–3.101)	0.895	1.439 (0.406–5.106)	0.573	1.312 (0.404–4.262)	0.651
N(2)	0.589 (0.137–2.523)	0.476	1.120 (0.241–5.205)	0.885	1.025 (0.246–4.270)	0.973
N(3)	0.847 (0.200–3.586)	0.822	2.117 (0.476–9.409)	0.325	2.022 (0.506–8.075)	0.319
Clinical stage		0.563		0.442		0.299
Clinical stage (1)	2.258 (0.297–17.150)	0.431	0.608 (0.037–9.896)	0.727	0.492 (0.058–4.183)	0.516
Clinical stage (2)	4.746 (0.335–67.150)	0.249	1.029 (0.037–28.548)	0.986	0.775 (0.054–11.212)	0.852
Clinical stage (3)	2.698 (0.095–76.460)	0.561	1.901 (0.060–60.743)	0.716	1.559 (0.092–26.538)	0.759

*LVI*, lymphovascular invasion; *PNI*, perineural invasion; *TDs*, tumor deposits

### Relationships between tumor deposits and TILs

**Fig. 5:**
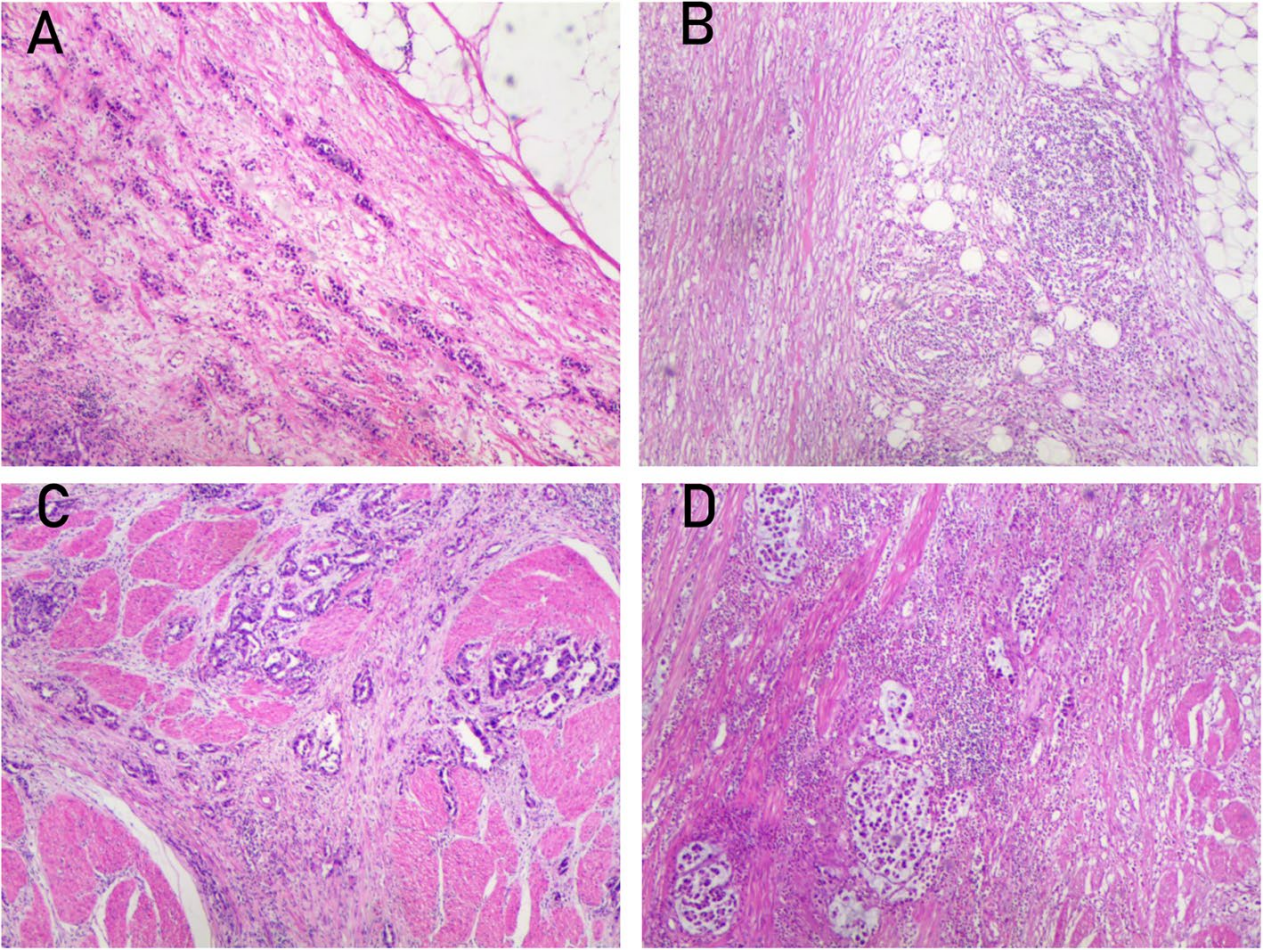
Expression of gastric cancer tumor-infiltrating lymphocytes. Expression of tumor-infiltrating lymphocytes. **A** and **C** Low-to-moderate tumor-infiltrating lymphocytes. **B** and **D** High tumor-infiltrating lymphocytes. (HE, hematoxylin and eosin × 40)

Among the 369 gastric cancer cases, 279 showed low-to-medium TILs (75.6%), and 90 were high TILs (24.4%) (Fig. 5). In the TDs(+) group, there were 74 cases with low-to-medium TILs and 7 cases with high TILs. In the TDs(−) group, there were 205 cases with low-to-medium TILs and 83 cases with high TILs. The differences between the two groups were statistically significant (*P* < 0.05) (Table 3).

**Table 3:** Correlation of tumor deposit and tumor-infiltrating lymphocytes

	TILs		
	Low-medium	High	*P*
TDs+	74	7	0.000
TDs−	205	83	

*TDs* tumor deposits, *TILs* tumor-infiltrating lymphocytes

## Discussion

This study analyzed the relationship between TDs and clinicopathologic characteristics and prognosis of gastric cancer. The results found that 22.0% of the 369 gastric cancer samples were TDs(+); TDs were significantly associated with gender, lymphovascular invasion, perineural invasion, pathological T,N and clinical stages, and significant survival differences between TDs(+) and TDs(−). TDs were an independent prognostic factor of DFS, CSS, and OS of gastric cancer. With the above basis in mind, we also studied the relationship between TDs and TILs in the tumor microenvironment, and the results found that the TDs were negatively related to the TILs, suggesting that there may be a complex relationship between TDs and tumor microenvironment; TDs and TILs may interact and affect the prognosis of patients with gastric cancer.

In 1935, Gabriel et al. firstly identified and reported TDs in colorectal cancer specimens, which were thought as the results of cancer cell dissemination along blood vessels [4]. The 8th edition of the AJCC/UNION for International Cancer Control defines TDs as discrete tumor nodules within the lymph drainage area of the primary carcinoma without identifiable lymph node tissue or identifiable vascular or neural structure [5]. In the pN staging of colorectal cancer, the absence of regional lymph node metastasis along with the presence of TDs within the subplasma and mesenteric tissues is classified as N1c. If both regional lymph node metastasis and TDs were present, the presence of TDs has no effect on staging, and the incidence of TDs in colorectal cancer ranges from 5 to 45% and was associated with a poor prognosis in colorectal cancer [13–15]. previous studies have found that TDs were presented not only in colorectal cancer but also in other solid malignancies, such as gastric, bile duct, and pancreatic cancers [2, 16]. Currently, although a few studies have shown that the presence of TDs were an independent prognostic factor for a poor prognosis in gastric cancer [9, 10], the mechanism of TDs formation were unclear. For colorectal cancer, the importance of TDs has been recognized and has been included in category N in the 7th edition of the TNM staging system for colorectal cancer. However, in the 8th edition of the TNM staging system, TDs were considered as a metastatic lymph node in gastric cancer, which is contrary to the findings of the current study. A recent retrospective study including 7445 gastric cancer cases showed that the incidence of TDs ranges from 10.6 to 36.7% (mean: 20.9%) [3]. Liang et al. studied 1034 gastric cancer patients with 240 (23.21%) TDs(+) and found that TDs were an independent prognostic factor for gastric cancer patients [2], which is similar to our findings. Therefore, the present study demonstrates that TDs were frequently observed and were an indicator of the aggressive characteristics of gastric cancer. The presence of TDs was a strong and independent prognostic factor and considered to be incorporated into staging strategies in gastric cancer.

Regarding the study on the number, size, and prognosis of TDs, Benoit et al. found that the number of TDs ≥ 4 had a lower DFS in rectal cancer [7]. In the present study, we investigated the relationships among the number of TDs, maximum diameter of TDs, and prognosis of gastric cancer. The results showed that the number of TDs was closely related to DFS, CSS, and OS of gastric cancer, and there was a significant difference in survival between the two groups. But in our study, the maximum diameter of TDs was not related to prognosis; this is similar to Raul’s study, suggesting that pathologists need to pay more attention to the number of TDs when observing the sections. The critical value of TDs should be verified by larger sample studies.

TILs are T lymphocytes, B lymphocytes, and NK cells that accumulate in the area of the tumor lesion and are at the forefront of the immune response and regulatory role in the tumor immune mechanism [17]. Studies have shown that the antitumor immune effect of TILs is mainly cellular, and dendritic cells present the major histocompatibility complex molecules of captured tumor neoantigens to T cells, leading to the activation of effector T cells and killing of tumor cells, which in turn secrete suppressive cytokines and have antitumor effects [18]; however, in the majority of cancer patients, the immune system fails to function effectively: it may be due to the failure of the immune system to recognize the tumor antigen and treat the tumor antigen as its own, i.e., immune tolerance, the inability of the effector T cells to infiltrate into the tumor lesion, or the suppressor (or immunosuppressive cells) in the tumor microenvironment inhibiting the function of effector cells [19]. In addition, the immune system, while removing tumor cells, also “reshapes” the characteristics of tumor cells to make them more malignant and more resistant to immune attack, i.e., “immune editing” [20]. Therefore, the immune system has a “double-edged sword” role in the process of tumor cell development, because TILs are a major player in tumor immunity, and there are many different subgroups of TILs, and the role of different subgroups in tumor development varies greatly, so the impact on tumor is also different [21]. In recent years, some researchers have pointed out that immune-related genes [22], Follistatin-like 1 (FSTL1) [23], immune T cell subgroup [24], preoperative neutrophil-lymphocyte ratio (NLR), and platelet-lymphocyte ratio (PLR) may be significantly associated with the prognosis of gastric cancer [25, 26]. The study shows that immune T cell subpopulation is closely related to the survival and recurrence rate of colorectal cancer and pancreatic cancer [27, 28]. Peripheral blood lymphocyte subpopulation predicts on the preoperative radiotherapy and chemotherapy response of rectal cancer [29]. Considering the occult pathogenesis of gastric cancer, its recurrence and metastasis are closely related to the tumor microenvironment. Further understanding of the role of tumor cells with the immune microenvironment in immunomodulation of gastric cancer is expected to improve current therapeutic strategies for gastric cancer.

In the present study, TDs have been shown to be an independent prognostic factor for gastric cancer patients, and TDs were associated with poor prognosis in gastric cancer, which is consistent with previous studies [9, 10]. And the present study also found that TDs were negatively correlated with TILs, and TILs levels were lower in TDs(+) group and higher in TDs(−) group. Therefore, in the tumor microenvironment of gastric cancer, TDs and TILs interact with each other to regulate the development of gastric cancer, thus affecting gastric cancer prognosis of patients. However, the mechanism of the interaction between TDs and TILs has not yet been elucidated, and more studies are needed to explore in the future.

However, there are a few limitations requiring further discussion. The findings of this retrospective study from a single Chinese institution may not be generalizable to other settings. Therefore, these findings should be considered only for hypothesis generation and require additional validation with more extensive studies.

## Conclusions

Our study found that TDs are significantly negatively correlated with TILs. TDs are independent predictors of the prognosis of gastric cancer, and the number of TDs ≥ 4 has a worse prognosis compared to the number of TDs < 4, which provides potential biological indicators for the diagnosis and treatment of gastric cancer and enriches the basis of antitumor immunotherapy.

## Data Availability

All analyses of the data have been reported in the Supporting Information File. In case any other clarification is needed, the relevant information will be made available with permission from the corresponding author.

## Abbreviations

AJCC: American Joint Cancer Committee
OS: Overall survival
CSS: Cancer-specific survival
DFS: Disease-free survival
TDs: Tumor deposits
TILs: Tumor-infiltrating lymphocytes
DFS: Disease-free survival
CSS: Cancer-specific survival
OS: Overall survival
